# Establishing the Callus-Based Isolation of Extracellular Vesicles from *Cissus quadrangularis* and Elucidating Their Role in Osteogenic Differentiation

**DOI:** 10.3390/jfb14110540

**Published:** 2023-11-02

**Authors:** Ritu Gupta, Sneha Gupta, Purva Gupta, Andreas K. Nüssler, Ashok Kumar

**Affiliations:** 1 Department of Biological Sciences and Bioengineering, Indian Institute of Technology Kanpur, Kanpur 208016, UP, India; ritu21@iitk.ac.in (R.G.);; 2 Siegfried-Weller Institute for Trauma Research, BG Trauma Center, University of Tuebingen, Schnarrenbergstrasse 95, 72070 Tuebingen, Germany; 3 Centre for Environmental Science and Engineering, Indian Institute of Technology Kanpur, Kanpur 208016, UP, India; 4 The Mehta Family Centre for Engineering in Medicine, Indian Institute of Technology Kanpur, Kanpur 208016, UP, India; 5 Centre of Excellence in Orthopaedics and Prosthetics, Gangwal School of Medical Sciences and Technology, Indian Institute of Technology Kanpur, Kanpur 208016, UP, India

**Keywords:** bone setter, callus tissue, *Cissus quadrangularis*, extracellular vesicles

## Abstract

Extracellular vesicles (EVs) are nano-sized vehicles secreted by all live cells to establish communication with adjacent cells. In recent years, mammalian EVs (MEVs) have been widely investigated for their therapeutic implications in human disease conditions. As the understanding of MEV composition and nature is advancing, scientists are constantly exploring alternatives for EV production with similar therapeutic potential. Plant-derived exosome-like nanovesicles (PDEVs) may be a better substitute for MEVs because of their widespread sources, cost-effectiveness, and ease of access. *Cissus quadrangularis* (CQ), known as “bone setter or Hadjod”, is a perennial plant utilized for its osteogenic potential. Its crude powder extract formulations are widely used as tablets and syrups. The present work elucidates the isolation of exosome-like nanovesicles (henceforth exosomes) from the culture supernatants of an in vitro cultured callus tissue derived from CQ. The physical and biological properties of the exosomes were successfully investigated using different characterization techniques. The therapeutic potential of the CQ exosomes was found to ameliorate the wound scratch injury and oxidative stress conditions in human-derived mesenchymal stem cells (hMSCs) and the pre-osteoblast (MC3T3) cell line. These exosomes also induced the proliferation and differentiation of hMSCs, as observed by alkaline phosphatase activity. These findings may serve as a proof of concept for further investigating the CQ exosomes as a nanocarrier for drug molecules in various therapeutic bone applications.

## 1. Introduction

Exosomes are small membrane-bound extracellular vesicles (30–200 nm) encased in a lipid bilayer. They play a pivotal role in intercellular communications and are regarded as potential delivery vehicles for diverse bioactive molecules [1]. These vesicles are present in all biological fluids and are secreted by all cells in the extracellular environment via the exocytosis of intraluminal vesicles. There was a long-standing belief that due to the presence of the cell wall, plants were incapable of producing extracellular vesicles (EVs). This notion was proven wrong in recent years, and it is now widely accepted that EVs can be released from any cell, including those of humans, animals, and even plants [2,3]. Although mammalian extracellular vesicles (MEVs) have shown promise in broad applications within the field of tissue engineering and regenerative medicine [4], their usage is surmounted by numerous challenges such as tissue specificity, toxicity, difficulty in isolation from biological fluids, and large-scale production costs [5,6]. Addressing these challenges, plant-derived extracellular vesicles (PDEVs) have emerged as cutting-edge, safe, eco-friendly, robust, and feasible carriers that can be cost-effectively produced in large amounts [7,8]. PDEVs are a suitable alternative for controlled and specific delivery of bioactive molecules [3].

Like MEVs, plants release exosome-like nanovesicles composed of bioactive lipids, nucleic acids, proteins, and small secondary metabolites into extracellular spaces. These vesicles take part in intercellular communications and serve as a biological defence against pathological conditions [9]. However, the chemical composition profile of PDEVs significantly differs from that of MEVs. PDEVs contain low concentrations of proteins predominantly of cytosolic origin (actin and proteolytic enzymes) and a few transmembrane proteins [10]. Notably, unlike transmembrane proteins such as tetraspanins, which are present in MEVs, PDEVs contain aquaporins and chloride channels as membrane proteins [11]. Whereas cholesterol and sphingolipids are the preliminary lipid components in mammalian vesicles, PDEVs are enriched in phospholipids and glycerol lipids. The lipid composition and vesicular contents of PDEVs vary from those of MEVs, implicating their vesicular roles and differential delivery within recipient human cells [12]. Furthermore, PDEVs contain miRNA, mRNA, and some unusual RNAs, among which miRNA regulates gene expression in recipient cells, affecting cell morphology and function [7].

PDEVs share a similar size and morphology with MEVs, and aside from fewer differences from MEVs, their biogenesis is conserved in PDEVs. Various studies have confirmed that PDEVs can be readily internalized into mammalian cells and result in diverse cellular responses, depending on the source and the vesicular cargo molecules [13,14]. These vesicles have also been explored as potential therapeutic agents in drug delivery systems for stomach and intestinal conditions [11], cancer treatment [15], pathogen vaccination [16], immunomodulation [17], and regenerative therapy [18]. Vesicles from plants are typically extracted from apoplastic fluid, plant juice, or homogenized whole plant, but they can also be obtained from the explant cultures such as callus and cell suspension cultures. The advantage of employing these explant cultures lies in their ability to produce secondary metabolites, their fast and continuous growth under sterile conditions, and in being the most native and reproducible source of EV production [19,20].

Several investigations have reported the therapeutic uses of the plant *Cissus quadrangularis* (CQ), commonly known as ‘Hadjod’ in the Indian Ayurvedic system. This plant is renowned for its fracture healing properties, antibacterial action, antioxidative effects, antiulcer treatment, antiosteoporotic benefits, cholinergic activity, gastroprotective action, and positive effects on cardiovascular activities [21]. Phytochemical analysis of methanolic extract has reported the presence of triterpenes such as α- and β- amyrins, ketosteroids, β- sitosterol, carotene, tannins, phenols, and vitamin C. Every part of this plant has been utilized in pharmacological actions, and is studied in the form of crude powdered extract for its effectiveness in therapies with a plant origin [22]. Even our research group has extensively studied the use of CQ extract in different bone applications [23,24]. 

In our present work, we hypothesized that CQ could serve as a source of EV isolation that can potentially induce cellular differentiation into the osteoblastic lineage. Accordingly, we initially established a plant-based in vitro culture system for callus induction from the leaf explants of CQ. We isolated and characterized CQ-derived extracellular nanovesicles (henceforth CQ exosome) from the culture supernatants. Further, we evaluated the osteogenic potential of hMSCs and myoblast (C2C12) cells in response to CQ exosomes. Our findings suggest that CQ exosomes have the ability to promote proliferation and differentiation of hMSCs and C2C12 cells to osteogenic lineage and may provide therapeutic benefits in osteoporosis and other bone degenerative diseases.

## 2. Materials and Methods

### 2.1. Materials

Whole plantlets of CQ were obtained from the Institute Nursery of the Indian Institute of Technology Kanpur. MS medium, MS-liquid medium, naphthylacetic acid (NAA), benzyl adenine (6-BAP), antibiotic cocktail (penicillin and streptomycin) and propidium iodide were purchased from HiMedia (Mumbai, India). 2-Methyl-1,4-naphthoquinone (menadione) was procured from the Tokyo Chemical Industry. Paraformaldehyde (PFA—98%) and vanillin were obtained from SD Fine Chem Ltd. Calcein AM and fetal bovine serum (FBS) were purchased from Gibco (MA). α-MEM, trypsin-EDTA, resazurin, β-glycerophosphate, BCA protein assay kit, alizarin red-S stain, ascorbic acid, and SIGMAFAST p-nitrophenyl phosphate were purchased from Sigma-Aldrich (St Louis, MO, USA). All additional chemicals used were of analytical grade. Human mesenchymal stem cells (hMSCs, PCS-500-010) and MC3T3-E1 (CRL-2593) cell lines were procured from ATCC, Manassas, VA, USA. Mouse myoblast cell line (C2C12) was procured from the National Centre for Cell Science, Pune, India.

### 2.2. Plant Material and Surface Sterilization

Whole plantlets of CQ were kept under maintenance for one month until new shoots and leaves were grown. Healthy leaf explants from the new shoots were selected for inoculation. After a vigorous surface sterilization, these explants were cut 8–10 mm in size. Fresh leaves were washed with running tap water for 10 min and then immersed in deionized water (DI) for 10 min to remove dirt. Further, the plant material was soaked in 0.5% sodium hypochlorite for 10 min, followed by 70% ethanol wash for 30 s, and then rinsed again with DI. Additionally, the explants were disinfected for 4 min with 0.1% mercuric chloride solution, rinsed several times with sterile DI, and kept for sectioning after drying.

#### 2.2.1. Cissus Cell Induction

Calli was induced from the sectioned leaf segments of CQ following the protocol, with some modifications, described elsewhere [25]. Briefly, the small leaf segments were incubated on a semi-solid MS medium supplemented with suitable hormonal concentrations of 3.36 mg/L naphthylacetic acid (NAA) and 1.5 mg/L benzyl adenine (6-BAP) as auxins and cytokinins, respectively. The pH of the medium was adjusted to 5.8 with 0.1 N NaOH/0.1 N HCl solution before being autoclaved for 20 min at 15 psi. Glutamine solution (15 mg/mL) was added to the media after autoclaving to increase the rate of callus induction. All the cultures were maintained at room temperature and subcultured at regular intervals under a 16 h day/light photoperiod with light intensity (150 µmol/m^2^/s) and 55–60% relative humidity in a plant tissue culture rack.

#### 2.2.2. Callus Suspension Culture and Growth Kinetics

Following 4–5 weeks of subculturing, actively proliferating CQ cells migrating from each callus were cultivated in liquid MS media to initiate a cell suspension culture. Briefly, 1 g of finely chopped calli was added to conical flasks containing 50 mL of MS liquid medium supplemented with 30 g/L sucrose and equal ratios of hormonal concentrations of auxins and cytokinins, as used earlier. A rotatory shaker (25 ± 2 °C, 110 rpm) was used to maintain the cultures in the dark and to produce cell suspension stocks for further inoculum [26]. After 15–17 days, fine cell suspension cultures were collected and used as an inoculum in each flask to determine the growth kinetics. The growth kinetics of the medium was measured as packed cell volume (PCV), and data were recorded every week as the percentage cell mass of the total centrifuged volume for 50 days, as reported [27].

#### 2.2.3. Cellular Extraction from the Callus

The suspension media was filtered using Whatman filter paper, the retentate was washed with DI, and then gently squeezed with filter paper to remove excessive water to extract callus material from the suspension culture flasks. The retentate (callus biomass) was then oven-dried for two days at 60 °C for dry weight determination (DW). Briefly, 100 mg of finely powdered (using mortar and pestle) dry sample was mixed with 1 mL of 80% methanol (*v*/*v*) as per the protocol described previously [28]. This mixture was kept at a constant shaking for 10 h at 37 °C, followed by sonication for 15 min and centrifugation for 10 min at 8000 rpm. The supernatant (referred to as whole-cell extract) was then collected and kept at 4 °C until needed [26].

### 2.3. Characterization of Callus and Biochemical Analysis of Cell Extract 

#### 2.3.1. Scanning Electron Microscopy

The surface morphology and interior structure of the calli were investigated using scanning electron microscopy (SEM). Briefly, the callus was cut into thin sections (roughly 5 mm × 5 mm) and desiccated overnight. Subsequently, these sections were fixed using 4% PFA and dehydrated with treatment of increasing ethanol gradient (20%, 40%, 60%, 70%, 90%, and 100%, 15 min incubation per ethanol concentration) followed by critical point drying in 100% ethanol and HMDS (1:2 ratio, respectively) for 10 min. The dried samples were first sputter-coated with gold for 60 s and imaged at a 10 kV accelerating voltage (Zeiss, Oberkochen, Germany, EVO 18) [29].

#### 2.3.2. Fourier-Transform Infrared Spectroscopy

The chemical structure of the whole-cell methanolic extract of CQ was studied using the Fourier-transform infrared spectrum (FTIR). The analysis was performed in the middle infrared range (4000–400 cm^−1^) using a PerkinElmer Spectrum Vision 10.03.06 FTIR spectrophotometer, as indicated before [30], and the results were compared with the leaf extracts of CQ to confirm the sample purity.

#### 2.3.3. Quantification of Total Phenolics

Total phenolic content of the whole-cell extract of CQ was determined using Folin–Ciocalteu reagent, following the protocol described elsewhere [31]. Gallic acid was the standard, dissolved in 80% (*v*/*v*) methanol. The calibration curve (0–10 g/mL, R^2^ = 0.9811) was used for the quantification, and the findings were displayed in mg gallic acid equivalent (GAE)/g of dry weight [26].

#### 2.3.4. Measurement of Antioxidant Activity by DPPH Assay

The antioxidant activity of the same extract was evaluated using the DPPH free radical scavenging test. Callus extract (50 mg) was treated with 100 μM solution of DPPH in methanol and incubated at 37 °C. Absorbance changes at 517 nm at various time points were used to determine the antioxidant capability. The antioxidant capacity was expressed using a previously established method [32]. The following equation was used to calculate the percentage of inhibition

% inhibition = (AB − AS)/AB × 100

where AS is the absorbance of the sample (DPPH + extract) and AB is the absorbance of the control (DPPH alone).

### 2.4. Exosome Isolation from CQ Cells

The filtrate (culture supernatant) from the cell suspension medium was collected to eliminate cell and cell debris through sequential centrifugation at 1000× *g* for 10 min and 3000× *g* for 20 min, respectively. The supernatant was then centrifuged using Oak Ridge tubes at 16,000× *g* for 45 min to remove microvesicles [9]. The proteins present in the supernatant were first separated based on their size, using a microfilter of pore diameter of 0.22 µm. The obtained protein solution was finally concentrated using 100 kDa Amicon ultrafilters (Millipore, Burlington, MA, USA) to obtain the nano-vesicle population containing exosomes [33]. The supernatant collected was resuspended in PBS and was stored at −80 °C till further use.

### 2.5. Characterization of CQ Exosomes

Different methods were used to assess the particle size, distribution, and morphology of CQ exosomes. These exosomes were also quantified and analysed to confirm their sample uniformity and successful isolation [34].

#### 2.5.1. Protein Content

Total protein concentration of the purified exosome sample was determined using a micro-BCA kit (ThermoFisher Scientific, Waltham, MA, USA) as per the manufacturer protocol. Protein concentration was calculated using the standard curve of BSA (0–250 µg/mL, R^2^ = 0.9947). 

#### 2.5.2. Lipid Content

Total lipid content of exosome suspension was estimated by sulfophosphovanillin (SPV) assay. Standard lipid solution was prepared in chloroform (2 mg/mL) consisting of cholesterol and potassium oleate (1:1). Briefly, 70 µL of standards was heated at 90 °C for 10 min to evaporate chloroform. After chloroform evaporation, 50 µL PBS for the standard and 50 µL suspension for the sample was added, followed by adding 96% H_2_SO_4_ (250 µL). This mixture was then heated at 90 °C for 20 min, and after being cooled to room temperature, 220 µL was added in a 96 well-plate. Further, vanillin (110 µL of 0.2 mg/mL) in 17% phosphoric acid was transferred to each well and kept undisturbed at room temperature for about 15 min. Finally, the absorbance at 540 nm was measured using a 200 µL solution [34].

#### 2.5.3. Dynamic Light Scattering

DLS analysis was performed to investigate the particle size distribution of exosomes. The exosome suspension was diluted in Milli-Q (1:50) to 1 mL and was placed in the Malvern Zetasizer ZS90. The zeta potential was measured thrice at 25 °C to evaluate the stability of exosomes.

#### 2.5.4. Scanning Electron Microscopy

SEM analysis was carried out to evaluate the morphology and size of the exosomes. The sample was diluted in a 1:1 ratio in 2% PFA for 6 h at 4 °C. Each dilution was made in Milli-Q water and drop-casted on a plasma-cleaned coverslip. The diluted samples were first sputter-coated with gold for 60 s and analysed using a scanning electron microscope (Zeiss, EVO 18) at 10 kV accelerating voltage. ImageJ software (version 1.54d) was used to measure the exosome particle size.

#### 2.5.5. Transmission Electron Microscopy

The morphology of exosomes was observed in high resolution using TEM. Initially, 2% PFA at room temperature was used to fix the exosome suspension for 15 min. The samples were mounted on 200-mesh-size copper grids and dried at room temperature. Image analysis was carried out using FEI- Tecnai G2 12 Twin TEM at an accelerating voltage of 120 kV, and the diameter of exosomes was studied using ImageJ software.

#### 2.5.6. Fourier-Transform Infrared Spectrum Analysis

FTIR spectroscopic analysis was used to determine biological macromolecular composition in the exosome suspension. The middle infrared region (4000–1000 cm^−1^) was used to record the spectra for the diluted samples, and the results were compared with mammalian exosomes (from hMSCs) to confirm the presence of similar constituents and sample uniformity, as shown previously [2].

### 2.6. In Vitro Cellular Studies of CQ Exosomes

#### 2.6.1. Exosome Internalization by hMSCs

An exosome internalization study was conducted on hMSCs utilizing labelled CQ exosomes. The exosomes were labelled using calcein AM (green) as per the protocol described, with some modifications [23,35]. Briefly, the hMSCs were cultured overnight on the gelatin-coated coverslips at a seeding density of 2 × 10^4^ cells/mL. Then, the CQ exosomes were incubated with calcein AM (1:100 dilution) for 30 min at 37 °C, followed by two subsequent 15 min cycles of ultrafiltration (Amicon ultrafilters 100 kDa) at 1000× *g*. These labelled exosomes were incubated with cells for 3 h for internalization, and the unbound exosomes were washed with warm PBS. The cells were then fixed and stained with TRITC-phalloidin and DAPI for observation under a confocal microscope (Leica SPII, Wetzlar, Germany).

#### 2.6.2. Cellular Viability under Oxidative Stress

The potential of CQ exosomes in response to oxidative stress was investigated, where MC3T3-E1 cells were exposed to oxidative stress after pre-treatment with exosomes. Cells were cultured in complete α-MEM media (α-MEM + 10% FBS + 1% antibiotic cocktail) in a humidified incubator at 37 °C, 95% humidity, and 5% CO_2_. After the cells reached 75–80% confluency, they were trypsinized (0.25% trypsin-EDTA), counted, and pelleted down. They were then seeded at a density of 1 × 10^4^ cells per well on a 48-well treated tissue-culture plate (TCP) and kept for 24 h for incubation. Cells were pre-treated with exosomes (12 µg/mL) in a serum-starved media for 6 h, and the same density of cells was seeded on a tissue-culture plate for the remaining groups in triplicates. One of the powerful ROS generators, menadione, causes oxidative stress-dependent cell death. Menadione concentration (20 µM) was chosen to induce reactive oxygen species (ROS) in the cells and assayed at different time points [36]. Three groups were chosen for the experiment (media, media + menadione, and media + menadione + exosomes). The cytotoxicity of cells was monitored over 8 h, quantitatively using lactate dehydrogenase (LDH) released by the cells and qualitatively with confocal microscopy at 4 h using live–dead imaging (calcein AM/PI). LDH, a cytosolic enzyme, is released into the media of the cell culture as a result of membrane disruption. Thus, the extent of extracellular LDH released into the medium is directly proportional to the cytotoxicity, which is then quantified using the LDH assay, as described earlier [33].

#### 2.6.3. Wound Scratch Assay

A wound scratch assay was performed to investigate the therapeutic potential of the exosomes, affecting the direction and rate of cell migration after tissue injury. Briefly, hMSCs at a density of 1 × 10^4^ cells per well were seeded on a 48-well treated TCP and were grown to 70% confluence for 12 h; the cells were then serum starved for 10 h. A micro scratch was made using a 10 µL tip, and the loosely adhered cells were detached using a PBS wash. Further, cells were treated with exosomes (12 µg/mL) and monitored over different intervals (0, 10, 20, 30 h). Imaging of the scratch was carried out with a phase-contrast microscope (Leica, DMi1, Germany), and ImageJ software was used to quantify the wound closure area [34].

#### 2.6.4. Cellular Proliferation in Response to Exosomes

In order to investigate the metabolic activity of cells in the presence of exosomes, a resazurin assay was performed [37]. hMSCs were cultured in T-25 flasks in α-MEM media (α-MEM + 10% FBS + 1% antibiotic cocktail) maintained in a humidified incubator at 37 °C. Cells were trypsinized once they attained the desired confluency, followed by centrifugation, resuspension, and cell counting. They were seeded at a density of 0.5 × 10^4^ cells/well in a 48-well TCP and were allowed to grow for 24 h. The cells were then treated with 40 µg/mL exosomes suspended in complete media. The media was replenished after every third day, including the fresh exosomal treatment, to maintain a constant concentration. In other groups, cells were treated with normal media as negative control and osteogenic induction medium (complete media + 10 mM β-glycerol phosphate + 50 μM ascorbic acid) as positive control. Resazurin solution (150 µL; 20 µg/mL) was added to each well and incubated at 37 °C in a CO_2_ incubator for 2.5 h. The readings were measured at 540/600 nm excitation/emission wavelength, and the assay was carried out at different time intervals (0, 1, 3, 7, and 14 d of culture). Using C2C12 myoblast cells, similar experiments were performed where cells were cultured in complete DMEM.

#### 2.6.5. Effect of CQ Exosomes in Cellular Differentiation

Cell differentiation potential was studied in hMSCs and C2C12 myoblast cells by determining alkaline phosphatase (ALP) activity. Alkaline phosphatase is an enzyme produced by osteoblasts in the early osteogenic phase and enables matrix mineralization. It utilizes p-nitrophenol phosphate (p-NPP) as a precursor, as described earlier [38].

Once the resazurin assay was performed, the same wells, in triplicate, were first washed with PBS, and then 100 µL of p-NPP substrate was added to each well and it was incubated for the next 30–45 min until p-nitrophenol, a yellow-coloured compound was obtained. The absorbance was measured at 405 nm [29], and the ALP activity was normalized with the total number of cells.

#### 2.6.6. Alizarin Red Staining

The osteogenic potential and mineralization of hMSCs were further investigated by staining the calcium nodules formed by the alizarin red assay. Alizarin red dye (pH 4.1) binds the calcium ions and helps locate the calcium ions produced by cells in the differentiated culture medium, thus colouring them red. A stock solution of dye with a 2 mg/mL concentration was prepared. This stock solution (150 µL) was added to each well after fixing the cells, followed by a PBS wash. After 1 h of incubation, the cells were washed thrice with warm PBS to remove the unbound dye and cellular debris. A bright-field microscope was used to image the stained crystals [36]. After imaging, 10% (*w*/*v*) cetyl pyridinium chloride solution was added to solubilize the bound calcium deposits of red precipitate, and it was kept undisturbed for 45 min under incubation until a colour change was observed, from red to purple. Finally, the developed absorbance was measured at 540 nm [23].

#### 2.6.7. Statistical Methods

All the experiments were performed in triplicate, and the data was reported as mean ± standard deviation (SD). The descriptive data analysis between groups was analysed using GraphPad Prism 8.0 software. All the experimental groups were compared using a two-way analysis of variance (ANOVA) with Tukey’s multiple comparison test. Statistically significant differences were presented at *p* < 0.0001 (****) with an alpha value of 0.05.

## 3. Results and Discussion

### 3.1. Callus Formation and Growth Curve

**Figure 1:**
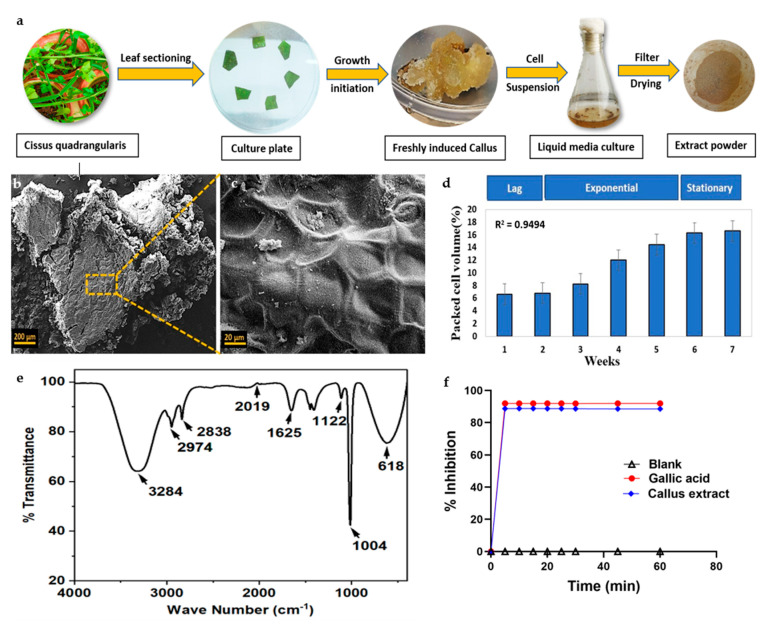
Characterization of leaf-induced callus and whole-cell extract of *Cissus quadrangularis*. (**a**) Schematic representation of freshly induced callus and isolation of whole-cell extract powder. (**b**,**c**) Representative scanning electron microscopic images showing CQ callus, scale bar: 200 µm and 20 µm, respectively. (**d**) Representation of cell growth in a suspension culture showing packed cell volume in relation to time for each of the growth stages (lag phase, exponential phase, linear phase, and stationary phase). (**e**) FTIR spectrum of CQ whole-cell extract powder showing major peaks. (**f**) DPPH assay demonstrating the antioxidant capacity of CQ whole-cell extract.

CQ leaf explants were inoculated with MS media enriched with suitable plant hormonal concentrations, resulting in successful induction of compact cream-coloured calli within 3 weeks, followed by brown-coloured friable calli formation in 6 weeks. The induced callus was subcultured at regular intervals of 30 days, by providing the same medium components and optimum growth conditions (Figure 1a).

The growth curve of the biomass produced by the CQ cell suspension culture showed a substantially slow rate of increase, with a 2-week initial lag period, immediately following a prolonged log phase for 5 weeks, and a subsequent stationary phase within the sixth week of the study, as depicted in Figure 1d. The maximum dry weight of the culture was found at the end of the 4 weeks (5 g/L). The biomass formed in the late log phase was then used for EV isolation and whole-cell extract formation (Figure 1a). 

### 3.2. Callus Characterization and Biochemical Evaluation of Cell Extract

#### 3.2.1. Scanning Electron Microscopy

SEM images revealed that the callus surface exhibited irregular amorphous outgrowths on the exterior, indicating the presence of a cell wall, as depicted in Figure 1b. The magnified images display the cellular network-like morphology and bridges that connect nearby cells (Figure 1c).

#### 3.2.2. Fourier-Transform Infrared Spectroscopy

FTIR analysis of the cellular extracts demonstrated the involvement of bioactive molecules and revealed the presence of compounds such as alcohol, aldehyde, isocyanides, nitrite, alkane, primary alcohol, and chloro constituent that demonstrated major peaks at 3284, 2974, 2838, 2019, 1625, 1122, 1004, and 618 cm^−1^, respectively (Figure 1e). These findings indicated the existence of phytochemicals such as alkaloids, tannins, carboxylic acids, flavonoids, and proteins within the whole-cell leaf extract of CQ. These results were also consistent with the major peaks involved in the original leaf exacts of CQ, as previously reported [39].

#### 3.2.3. Antioxidant Activity

Total phenolic content (1.43 mg gallic acid equivalent (GAE/g) of dry weight) and antioxidant activity (88.6%) were found in the stationary phase of the suspension culture that was 40 days old, as shown in Figure 1f. All the experiments were performed in triplicate and showed a favourable correlation between phenolic constituents and antioxidant potential in CQ callus cultures.

### 3.3. Isolation of Exosomes

**Figure 2:**
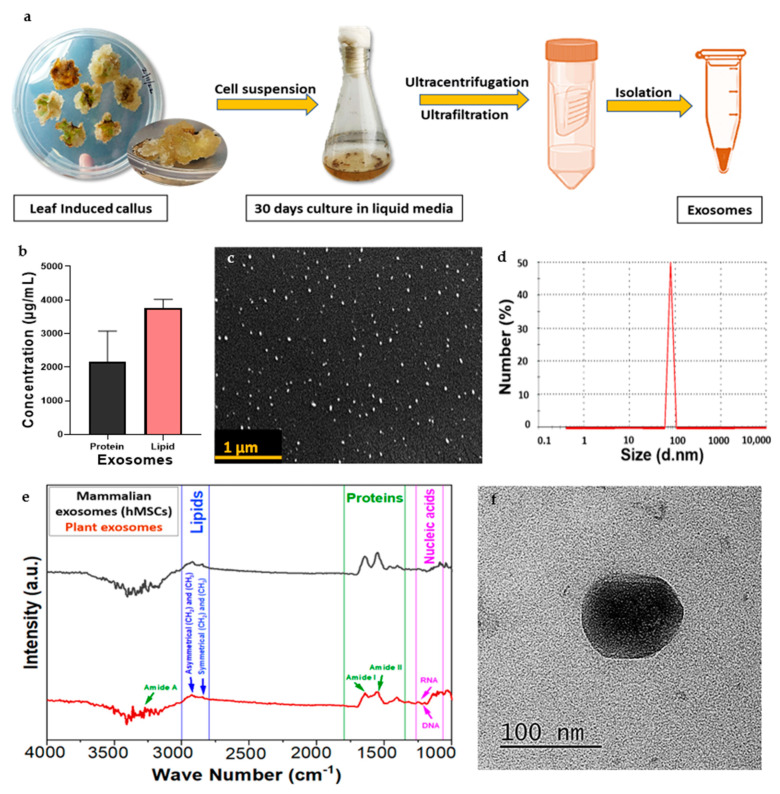
Characterization of Cissus-derived extracellular vesicles (CQ exosomes). (**a**) Schematic representation of exosome isolation from the filtrate of suspended media extracted from leaf-induced callus of CQ. (**b**) Estimation of total protein and lipid concentration in the exosomes. (**c**) Representative scanning electron microscopy image showing the morphology of exosomes, scale bar: 1 µm. (**d**) Size distribution of exosomes using dynamic light scattering. (**e**) Comparison between FTIR spectra for biological compositions of plant-derived and mammalian exosomes. (**f**) Representative transmission electron microscopy image showing the morphology of exosomes, scale bar: 100 nm.

During its growth, exosomes were released from the CQ callus in the cell suspension media. CQ exosomes were successfully isolated from the filtrate of the suspension media using ultrafiltration and ultracentrifugation, as depicted in Figure 2a.

### 3.4. Characterization of CQ Exosomes

#### 3.4.1. Estimation of Protein and Lipid Content

The protein concentration of the obtained CQ exosomal solution was around (1280 ± 775.8 µg/mL), and that of the lipid was (3866 ± 251.6 µg/mL), as illustrated in Figure 2b. In terms of lipid-to-protein ratio, the purity of CQ exosomes was found to be 3%, which differs from MEVs, i.e., 2–2.5% [1]. This difference suggests that higher lipid levels in plant-derived vesicles may effectively improve cellular intake and efficiently deliver bioactive materials to the target cells [12]. 

#### 3.4.2. Dynamic Light Scattering

The vesicles showed an average diameter of 79.25 ± 8.22 nm with a mean polydispersity index of 0.3 ± 0.03, which indicates that the isolated nano-range extracellular vesicles (exosomes) were heterogenous (Figure 2d). The zeta potential was around −17.5 ± 11.81 mV, indicating that the particles were negatively charged. 

#### 3.4.3. Scanning Electron Microscopy

Exosomes displayed a closed spherical structure, as seen in Figure 2c, with a size range of 84.49 ± 9.27 nm. These findings were consistent with the dynamic light scattering data. The analysis showed that the exosomes were evenly distributed and did not aggregate.

#### 3.4.4. Transmission Electron Microscopy

The TEM analysis also confirmed the spherical-shaped vesicular structure of exosomes with visible boundaries. The diameter of exosomes was calculated as 86.77 ± 7.2 nm, as shown in Figure 2f. This average particle size range was consistent with other characterization techniques.

#### 3.4.5. Fourier-Transform Infrared Spectrum Analysis

Exosomes isolated from culture supernatants of CQ contain biologically active macromolecules such as nucleic acids, lipids, proteins, and small metabolites. The band area for different biomacromolecules was divided into four categories: (1) amide I (1674–1627 cm^−1^), (2) amide II (1557–1509 cm^−1^), (3) lipids (2972–2844 and 1754–1736 cm^−1^, and (4) nucleic acids (1267–1215 and 1124–1066 cm^−1^), as depicted in Figure 2e. The major peaks found in this study were compared with MEVs (hMSCs) to confirm the presence of similar bioconstituents.

### 3.5. In Vitro Cellular Studies of CQ Exosomes

#### 3.5.1. Exosome Internalization

**Figure 3:**
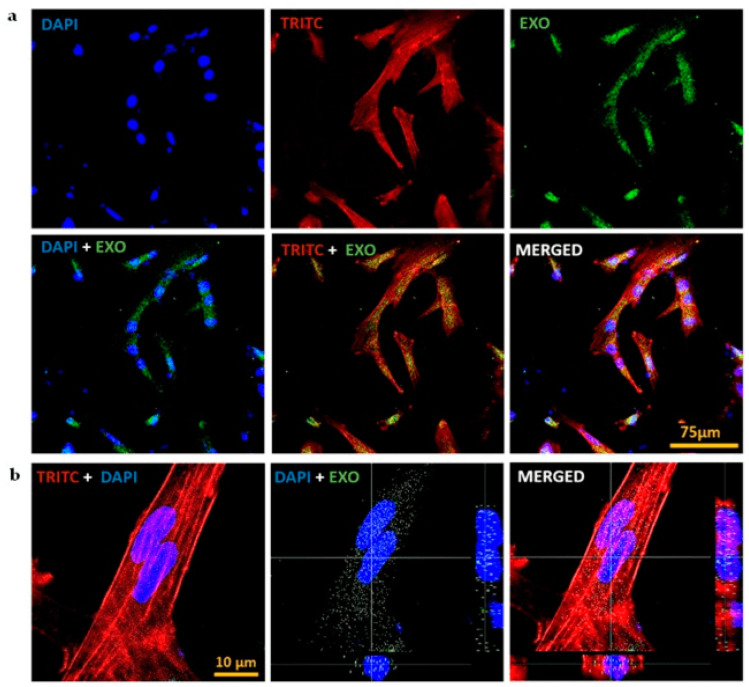
Internalisation of CQ exosomes. (**a**) CLSM of internalization of exosomes by hMSCs after labelling exosomes with calcein AM, the cytoskeleton with phalloidin (TRITC), and the nucleus with DAPI, scale bar: 75 µm. (**b**) Orthogonal sectioning of confocal images representing the fact that exosomes are present inside the cell and are located near the nucleus of the cells, scale bar: 10 µm.

PDEVs are nano-sized vesicles with complex biomolecules that deliver signals to target cells by their easy internalization into mammalian cells. Many studies have shown that these PDEVs evoke distinct effects on the functionality of various recipient cell types [40]. To visualize their uptake, we studied the internalization of CQ exosomes by hMSCs after labelling them with calcein AM. The hMSCs cytoskeleton was stained with phalloidin (TRITC) and the nucleus with DAPI. The results indicated the successful internalization of exosomes, as depicted in Figure 3a. To further conclude the results, the orthogonal sectioning of the images was obtained, which showed that the exosomes are present inside the cell mostly near the nucleus, rather than on the surface Figure 3b.

#### 3.5.2. Cellular Viability under Oxidative Stress

**Figure 4:**
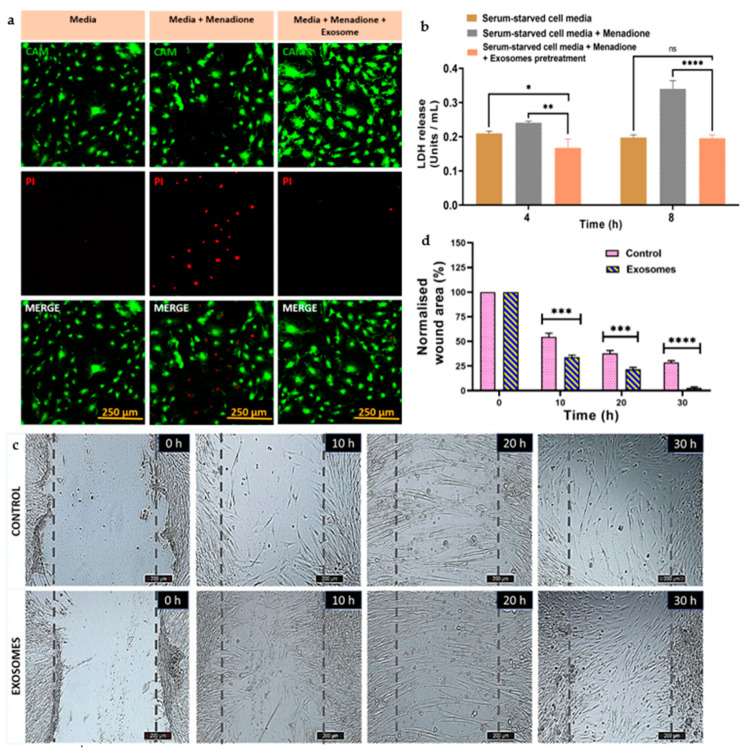
Antioxidant and cell migratory properties of CQ exosomes. (**a**) Effect of exosomes on MC3T3 cells under oxidative stress by menadione treatment (20 µM) at 4 h, scale bar: 250 µm, showing that cells were able to attenuate the oxidative stress in the presence of exosomes and preserve their healthy morphology and viability. (**b**) Effect of exosomes on LDH activity at different time points, i.e., 4 and 8 h. (**c**) Representative bright-field microscopic images of hMSCs migration with or without exosome treatment at different time intervals, i.e., 0, 10, 20, 30 h, scale bar: 200 µm. (**d**) Quantitative analysis of the microscopic images expressed as percentage normalized wound area. Data represented as mean ± s.d. (ns: non-significant, * *p* ≤ 0.05, ** *p* ≤ 0.01, *** *p* ≤ 0.001, **** *p* ≤ 0.0001).

The viability of MC3T3-E1 cells was analysed using calcein AM/PI fluorescence labelling in the presence of a strong antioxidant, i.e., menadione. Menadione accumulates superoxide within the cell through mitochondrial enzymes and causes a reduction in the antioxidant activity of the cell, which eventually induces cell death [41]. The cells were pre-treated with or without exosomes, and the effects on cells were seen qualitatively for 4 h. We observed that more live cells (green fluorescence) were present in the exosomal pre-treated group in comparison to the non-exosomal pre-treated groups. As depicted in Figure 4a, cells treated with exosomes were able to reduce oxidative stress, preserving their healthy morphology and viability when compared to other groups. Thus, the exosomes were able to attenuate the oxidative stress generated. This observation can be attributed to the strong antioxidant effect of exosomes, which they show by increasing the levels of superoxide dismutase, catalases, and glutathione [42].

Furthermore, the quantitative analysis was performed using LDH assay to confirm the metabolic activity and the attenuation of cellular death in response to exosomes. Figure 4b graphically represents the fact that serum-starved menadione-treated cells seeded on a tissue culture plate released more LDH into the medium, indicating higher cell death. However, LDH activity significantly decreased when serum-starved cells were treated with both menadione and exosomes, in contrast to cells treated with medium alone. This demonstrated that in the presence of exosomes, cells were metabolically active and exhibited higher cellular viability.

#### 3.5.3. Wound Scratch Assay

To mimic the cellular migration upon any tissue damage or injury, a wound scratch assay was performed with hMSCs. Bright-field microscopy was used to capture the images showing how the hMSCs migrated at various time points for 30 h, as indicated in Figure 4c, in both the presence and absence of exosomes. In the exosome-treated group, the cells filled the scratch area as they started migrating towards each other, but in the control group (where no treatment was provided), a significant portion of the wound area remained open. Quantitative analyses of the % wound area, as seen in Figure 4d, indicated that after 10 h, around 60% of the scratch area remained open in the control group, whereas 31% was left in the exosome group. Even after 30 h of the experiment, about 27% of the wound in the control group was open, whereas 100% of the area was closed in the exosome group. These results confirmed that in the presence of serum-starved medium containing exosomes, cells migrate faster than cells containing serum-starved medium alone. The results suggest the ability of CQ exosomes to regenerate damaged or injured tissue by facilitating the migration of native cells. 

#### 3.5.4. Cellular Proliferation in the Presence of Exosomes

**Figure 5:**
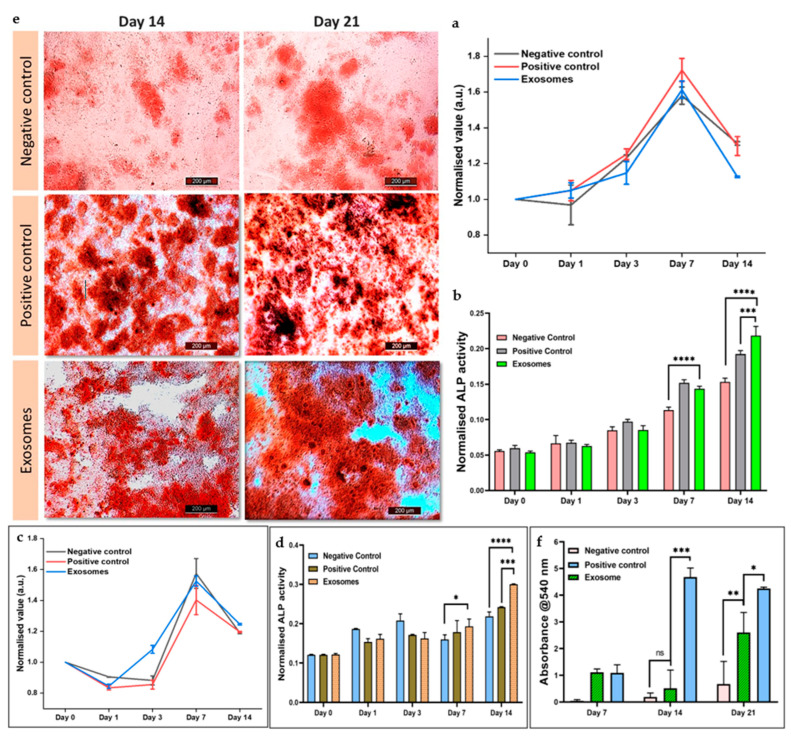
Effect of exosome on viability and differentiation of hMSCs and C2C12 cells. (**a**) Cellular metabolic activity of hMSCs, as quantified by the resazurin assay, shows that cells proliferate well in the presence of exosomes. (**b**) Alkaline phosphatase assay (an early osteogenic differentiation marker) of hMSCs. (**c**) Cellular metabolic activity of C2C12 cells represents the fact that the cells proliferated well in the presence of exosomes. (**d**) Alkaline phosphatase assay of C2C12 cells showed that in response to exosomes, cells could differentiate in the osteogenic lineage. (**e**) Effect of exosomes on the mineralization of hMSCs through alizarin staining (negative control: normal media; positive control: normal media + osteogenic induction media; exosomes: normal media + exosomes), showing that in the presence of exosomes, hMSCs differentiate to osteogenic lineage by forming calcium nodules, scale bar: 200 µm. (**f**) Quantification of mineralized nodules using alizarin red indicates that the exosomes can increase the osteogenic expression in hMSCs. Data represented as mean ± s.d. (ns: non-significant, * *p* ≤ 0.05, ** *p* ≤ 0.01, *** *p* ≤ 0.001, **** *p* ≤ 0.0001).

The cellular viability of both cell types (hMSCs and C2C12 cells) in response to CQ exosomes was assessed using the resazurin assay. Three groups, each in triplicate, were selected for the experiment: negative control, positive control, and exosome group. Figure 5a showed that by day 3, cellular metabolic activity in all groups began to increase and remained consistent until day 7. At day 7, the number of viable cells was significantly higher in all the groups, depicting the fact that the exosomes did not induce cytotoxicity. However, the analysis shows that at later time points (day 14), cellular viability decreased as compared to day 7, possibly due to the space constraints in the well plate. However, the cellular activity was consistent among all groups at day 14, indicating no deleterious effects due to exosomes.

Similar results are depicted in Figure 5c from when C2C12 cells were cultured in DMEM.

#### 3.5.5. Cellular Differentiation in Response to Exosomes

The osteogenic capacity and differentiation of hMSCs and C2C12 cells were assessed using an alkaline phosphatase assay. In this experiment, the same treatment groups as in the resazurin assay were employed. The osteogenic potential of CQ exosomes was comparable to the positive control and much higher than the negative control after 7 days, as shown in Figure 5b. Furthermore, after 14 days, ALP activity increased significantly in exosomes compared to the negative and positive control. The results demonstrated that hMSCs differentiate to osteogenic lineage in response to CQ exosomes. Similar results are observed in Figure 5d, where C2C12 cells were cultured in DMEM. The ALP expression in the CQ exosome group in 14 days was significantly higher than in the negative control group (media alone) and the positive control group (osteogenic induction medium). 

Many studies have documented the usage of these cells, i.e., human mesenchymal stem cells (hMSCs) and mouse myoblast cells (C2C12), to study the osteogenic differentiation pattern in response to certain osteogenic differentiation factors such as Runx2 and BMP2, respectively [43,44]. Thus, we have used these cells to investigate the osteogenic potential of the obtained microvesicles for potential bone tissue engineering applications. Therefore, it may be due to the presence of these osteogenic differentiation factors that facilitated the differentiation of cells in the presence of CQ exosomes.

#### 3.5.6. Alizarin Red Staining for Calcium Deposition

Alizarin red is a late marker for osteogenesis. The osteogenic differentiation and mineralization of hMSCs in response to CQ exosomes were visualized by staining the generated calcium nodules with alizarin red staining (at day 7, 14, and 21) and compared with the positive and negative controls (Figure 5e). Subsequently, these alizarin red-stained calcium nodules were extracted and represented graphically. We found that the number of mineralized nodules deposited in the exosomal group was significantly higher than in other groups on day 14 and day 21, as depicted in Figure 5f. We speculate that these exosomes may have enhanced the expression of osteoblast differentiation markers and other important cues that demonstrate potential application in bone regeneration strategies [44]. 

## 4. Conclusions

The study highlights the fact that an in vitro tissue culture system can offer rapid and continuous growth under sterile conditions and provide a reproducible source of plant-derived extracellular vesicles (PDEVs) for drug delivery in therapeutic applications. PDEVs provide several advantages over mammalian EVs in terms of safety, biocompatibility, scalability, and cost-effective large-scale production. Our data demonstrated the significant role of CQ exosomes in terms of their antioxidative, cellular migration, proliferation, and osteogenic differentiation abilities. These findings underscore the therapeutic utility of these exosomes, derived from the bone-healing plant, and potentiate their importance in bone tissue engineering.

## Data Availability

The data presented in this study are available on request from the corresponding author.
